# Versatile Three-Dimensional Head and Neck Reconstruction Using a Thoracodorsal Artery-Based Chimeric Flap: A Bi-Institutional Experience

**DOI:** 10.3390/jcm15062398

**Published:** 2026-03-21

**Authors:** Youn Hwan Kim, Seung Eun Hong, Daihun Kang

**Affiliations:** 1 Department of Plastic and Reconstructive Surgery, College of Medicine, Hanyang University, Seoul 04763, Republic of Korea; younhwank@hanyang.ac.kr; 2 Department of Plastic and Reconstructive Surgery, Ewha Womans University Seoul Hospital, Seoul 07804, Republic of Korea; monkeyhong@ewha.ac.kr; 3 College of Medicine, Ewha Womans University, Seoul 07804, Republic of Korea

**Keywords:** head and neck reconstruction, chimeric flap, thoracodorsal artery perforator flap, latissimus dorsi, free flap

## Abstract

**Background**: Complex head and neck defects often require simultaneous reconstruction of multiple tissue types. The thoracodorsal artery-based chimeric flap offers the potential to address these requirements through a single vascular pedicle. **Methods**: A retrospective review of patients who underwent head and neck reconstruction using thoracodorsal chimeric flaps at two institutions (2009–2026) was performed. Flap configurations incorporated combinations of the thoracodorsal artery perforator skin paddle, latissimus dorsi muscle, and serratus anterior muscle. **Results**: Nineteen patients (mean age 63.2 years) were included. Primary sites were the hypopharynx (42.1%) and oral cavity (36.8%). Flap survival was 100%. Reconstruction-related complications occurred in 47.4% of patients, most commonly pharyngocutaneous fistula or leakage (31.6%), all managed conservatively or with secondary closure. Among survivors, 100% achieved tracheostomy decannulation and oral intake. **Conclusions**: The thoracodorsal chimeric flap may be a useful option for complex head and neck reconstruction requiring multiple tissue components through a single pedicle. However, the complication rate highlights the challenges inherent in this high-risk population, warranting further prospective validation.

## 1. Introduction

Head and neck reconstruction following oncologic ablation presents unique challenges that distinguish it from reconstruction in other anatomical regions. While extremity reconstruction typically involves two-dimensional tissue replacement with a single tissue type, head and neck defects frequently demand both a three-dimensional spatial approach and the provision of multiple, functionally distinct tissue components [1,2]. A single defect following ablative surgery for oral, pharyngeal, or maxillary malignancy may simultaneously require thin pliable skin for mucosal lining, additional skin or muscle for external wound coverage, muscle bulk for dead space obliteration, and occasionally bony framework for structural support [3]. Success in these cases is defined not merely by flap survival, but by the restoration of critical functions including swallowing, speech, and airway protection, as well as acceptable aesthetic outcomes [4,5].

For complex composite defects, two reconstructive strategies have traditionally been employed: the use of two separate free flaps, or the use of a chimeric flap in which a single vascular pedicle supports multiple tissue components [6]. The double-flap approach offers greater flexibility in flap positioning and can effectively address large, multidimensional defects. However, this strategy substantially increases operative time, requires two sets of microvascular anastomoses, consumes multiple recipient vessels, and imposes a significant physiological burden on patients who are often elderly and medically compromised.

Chimeric flaps offer an elegant solution to these limitations by providing multiple tissue components through a single vascular pedicle [7]. The anterolateral thigh (ALT) chimeric perforator flap has gained widespread popularity for head and neck reconstruction due to its versatility and acceptable donor site morbidity [8]. However, the ALT system is constrained by the inherent variability of its perforator anatomy [9]. Multiple sizable perforators capable of independently supporting separate flap components are infrequently encountered [10]. When the ALT flap is divided into separate paddles for simultaneous intraoral and external coverage, the risk of distal flap necrosis increases, and intraoperative planning becomes unpredictable [11].

The thoracodorsal vascular system presents a compelling alternative. The thoracodorsal artery demonstrates highly consistent anatomy, with predictable branching to the latissimus dorsi muscle and reliable angular branches to the serratus anterior muscle [12,13]. This anatomical consistency enables the design of chimeric flaps incorporating multiple independent tissue components—including the thoracodorsal artery perforator (TDAP) skin paddle, latissimus dorsi muscle, and serratus anterior muscle—all perfused by a single vascular pedicle [14]. Furthermore, the thoracodorsal chimeric flap can be harvested in the supine position, facilitating a simultaneous two-team approach with the ablative surgical team and thereby reducing total operative time [15].

Despite these theoretical advantages, systematic case series describing the specific chimeric combination of the TDAP skin paddle, latissimus dorsi muscle, and serratus anterior muscle for head and neck reconstruction remain scarce [15]. Existing reports have primarily focused on the TDAP as a single flap or on TDAP-scapular tip configurations, and the clinical utility of the multi-component soft tissue chimeric approach across diverse head and neck defect types has not been comprehensively evaluated. The purpose of this study is to present a bi-institutional experience with the thoracodorsal chimeric flap system for complex head and neck reconstruction, describing the surgical technique, a recipient vessel selection strategy, a structured comparison with alternative reconstructive options, and evaluating clinical outcomes with their limitations.

## 2. Methods

### 2.1. Study Design and Patient Selection

This retrospective case series was conducted at two tertiary academic medical centers (Institution A and Institution B) in South Korea. Patients who underwent head and neck reconstruction using a thoracodorsal artery-based chimeric flap were identified through a review of surgical databases and medical records. The study period was February 2009 to January 2026 at Institution A, and December 2024 to January 2026 at Institution B.

Inclusion criteria were: (1) complex head and neck defects requiring three-dimensional reconstruction following oncologic resection or salvage surgery, and (2) use of a chimeric flap based on the thoracodorsal vascular system, including combinations of the thoracodorsal artery perforator (TDAP) skin paddle, latissimus dorsi (LD) muscle, and serratus anterior (SA) muscle. Patients who underwent reconstruction with non-thoracodorsal system flaps (e.g., anterolateral thigh flap, radial forearm flap) were excluded.

This study was approved by the Institutional Review Boards of both institutions (IRB No. 2026-01-018 and SEUMC IRB 2026-01-048-001). The requirement for informed consent was waived due to the retrospective nature of the study.

### 2.2. Preoperative Evaluation

All patients underwent comprehensive preoperative assessment, including computed tomography (CT) or magnetic resonance imaging (MRI) of the head and neck, chest radiography, and laboratory workup. Tumor staging was performed according to the American Joint Committee on Cancer (AJCC) staging system. Patients’ prior treatment history, including radiotherapy, chemotherapy, and previous surgery, was recorded as part of the preoperative assessment.

### 2.3. Surgical Technique

All reconstructive procedures were performed by the same senior surgeon (Y.H.K.) across both institutions under general anesthesia. The operative workflow consisted of three sequential phases: flap design and harvest, recipient vessel preparation with microvascular anastomosis, and flap inset. The reconstructive team entered the operative field as the head and neck surgery team was completing tumor ablation and neck dissection. Flap harvest was typically initiated during the final stages of the ablative procedure to minimize total operative time. The chimeric flap was designed preoperatively based on the individual defect characteristics, and the specific combination of flap components was determined according to the reconstructive requirements of each case.

#### 2.3.1. Flap Design and Harvest

The patient was placed in a supine position with the ipsilateral arm abducted and elevated. An incision was made along the midportion between the anterior border of the LD muscle and the pectoralis major muscle. The anterior border of the LD muscle was identified, where numerous perforators are typically located. Once one or two reliable perforators were confirmed and incorporated into the skin paddle design, intramuscular dissection was carried out along the perforator course toward the thoracodorsal vessels, with care taken to preserve the thoracodorsal nerve.

We routinely employed a muscle-sparing technique, preserving a muscle cuff approximately 8–10 cm in diameter at the bifurcation of the descending and transverse branches of the thoracodorsal vessels. Because it is technically demanding to include the LD muscle itself as a chimeric component during muscle-sparing harvest, a two-paddle chimeric flap configuration incorporating the serratus anterior (SA) muscle was utilized when an additional muscle component was required. This muscle-sparing approach was particularly indicated for reconstructions requiring a large skin paddle with substantial soft-tissue volume.

The LD muscle was harvested with the muscular branches arising from the descending or transverse branch of the thoracodorsal vessels. The SA muscle was also harvested as needed. Vascular branches that did not supply the flap components were ligated to obtain a longer pedicle length. During ligation, the transverse branch of the thoracodorsal nerve was preserved to maintain donor-site function; however, ligation of the descending branch of the thoracodorsal nerve was unavoidable in certain cases. After confirming adequate pedicle length and vessel caliber, the skin flap was elevated in a caudal-to-cephalic direction.

The flap was designed based on the defect requirements, incorporating one or more of the following components:

- TDAP skin paddle: A fasciocutaneous component based on perforators from the descending or transverse branch of the thoracodorsal artery, used for mucosal lining or external skin coverage.
- Latissimus dorsi muscle: A partial segment for dead space obliteration, volume replacement, or reinforcement of mucosal lining sutures.
- Serratus anterior muscle: Used for supplementary volume augmentation, reinforcement of the mucosal lining, or protection of exposed vessels following neck dissection and tumor ablation.

In cases requiring bony reconstruction, non-vascularized fibula or iliac bone grafts were harvested and incorporated into the chimeric flap design. Vascularized osseous reconstruction was not the primary objective of this study, which focused on evaluating the versatility of the thoracodorsal chimeric flap system for soft tissue–dominant composite head and neck defects.

#### 2.3.2. Recipient Vessel Selection and Microvascular Anastomosis

**Figure 1:**
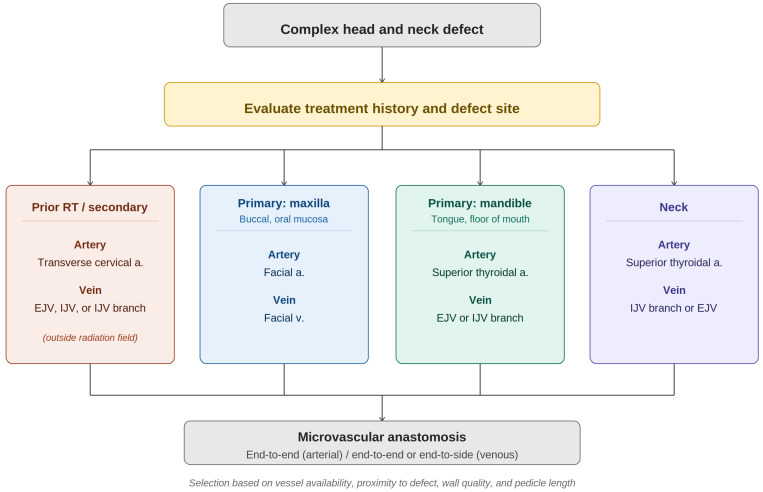
Algorithm for recipient vessel selection in thoracodorsal artery-based chimeric flap reconstruction. Vessel choice is guided by prior radiation history and anatomical location of the defect. RT = radiation therapy; EJV = external jugular vein; IJV = internal jugular vein.

Recipient vessels were selected based on availability and proximity to the defect. The superior thyroid artery was most commonly used for arterial anastomosis. Venous drainage was established using the internal jugular vein, external jugular vein, or facial vein branches. Arterial anastomoses were performed in an end-to-end fashion, while venous anastomoses were performed in an end-to-end or end-to-side fashion, under microscopic magnification. The recipient vessel selection strategy, incorporating defect location and prior treatment history, is summarized in Figure 1.

#### 2.3.3. Flap Inset and Layered Closure

The chimeric configuration allowed independent positioning of each flap component. The skin paddle was inset for mucosal or cutaneous reconstruction, while the muscle components were tailored to fill dead space, protect vital structures, or reinforce areas at risk of fistula formation. To minimize the risk of pharyngocutaneous fistula, a multi-layered closure technique was routinely employed. The mucosal lining was first closed with skin sutures, followed by a second layer of dermal sutures, and finally reinforced by overlying the muscle component as a third protective layer, creating a robust seal between the pharyngeal lumen and the surrounding tissues.

### 2.4. Postoperative Management

Patients were monitored in the intensive care unit for the first 24–48 h. Flap viability was assessed clinically by evaluating color, temperature, and capillary refill. A tracheostomy was maintained until airway patency and swallowing function were confirmed. Oral intake was initiated after confirming the absence of salivary leakage, typically within 3–4 weeks postoperatively.

Particular attention was given to protecting the intraoral flap from salivary contamination and oral secretions. Frequent suctioning of the oral cavity was performed to minimize the accumulation of secretions around the flap. Once the patient’s condition permitted, a structured oral care regimen was implemented, including regular gargling and gentle irrigation of the oral cavity. These measures were considered essential in reducing the risk of local infection and promoting undisturbed flap healing in the inherently contaminated intraoral environment.

### 2.5. Data Collection and Outcome Measures

The following variables were collected: patient demographics, primary diagnosis, tumor staging, prior treatment history, flap composition, pedicle length, recipient vessels, operative complications, functional outcomes (tracheostomy decannulation, oral intake), and follow-up duration. Flap-related complications were defined as any adverse event directly attributable to the reconstructed flap, including partial necrosis, dehiscence, infection, or fistula formation. Due to the descriptive nature and limited sample size, no comparative statistical analysis was performed.

## 3. Results

### 3.1. Patient Characteristics and Follow-Up

A total of 19 patients (14 males and 5 females) were included in this study. The mean age was 63.2 ± 11.2 years (range, 42–82 years). The most common primary site was the hypopharynx (*n* = 8, 42.1%), followed by the oral cavity (*n* = 7, 36.8%) and larynx (*n* = 2, 10.5%). Four patients (21.1%) underwent salvage reconstruction for recurrent disease or failed previous treatments. The mean follow-up duration was 9.8 ± 8.3 months, ranging from 2 to 36 months. Patient demographics are summarized in Table 1 and detailed clinical data for each case are provided in Table 2.

### 3.2. Reconstructive Details and Flap Anatomy

All 19 cases were successfully reconstructed using the thoracodorsal artery-based chimeric system. The mean pedicle length was 7.5 ± 1.3 cm, providing sufficient reach for anastomosis to various recipient vessels, primarily the superior thyroid artery (57.9%).

The flap design was tailored to each defect’s three-dimensional requirements. The most common configuration was a TDAP skin paddle combined with an LD muscle segment (*n* = 11, 57.9%), followed by an LD muscle-sparing or musculocutaneous skin paddle combined with an SA muscle segment (*n* = 5, 26.3%), and a TDAP skin paddle combined with an SA muscle segment (*n* = 2, 10.5%). Overall, the LD muscle was utilized in 17 cases (89.5%)—either as an independent muscle component or as the vascular basis for the skin paddle—and the serratus anterior muscle in 8 cases (42.1%).

A “three-paddle” configuration incorporating all three components—the TDAP skin paddle, LD and SA muscle—was employed in one patient for pharyngolaryngeal reconstruction requiring simultaneous mucosal restoration, carotid artery coverage, and sternocleidomastoid muscle replacement. In one case requiring a large skin paddle for extensive parotid defect coverage, supercharging through an additional anastomosis to the transverse cervical vessels was performed to ensure adequate flap perfusion. In three cases of extensive bony defects (maxilla and mandible), non-vascularized bone grafts were integrated and wrapped with the muscle component to provide a vascularized biological envelope.

### 3.3. Surgical and Functional Outcomes

The overall flap survival rate was 100% (19/19), with no total or partial necrosis reported. Reconstruction-related complications occurred in 9 patients (47.4%), with a total of 11 complication events observed, as two patients experienced multiple complications. Pharyngocutaneous fistula or minor salivary leakage was the most frequent complication (*n* = 6, 31.6%), predominantly occurring in pharyngeal and laryngeal reconstruction cases involving mucosal suture lines. These were successfully managed with conservative wound care or secondary closure. Other complications included wound dehiscence (*n* = 2, 10.5%), seroma or hematoma (*n* = 2, 10.5%), and localized wound infection (*n* = 1, 5.3%).

Functional recovery was assessed by the restoration of oral intake and tracheostomy decannulation. Among the 14 surviving patients, 100% achieved successful decannulation and transitioned to a soft or regular diet. The average time to initiate oral intake was 3.4 weeks postoperatively. The thin, pliable nature of the TDAP skin paddle allowed for subjectively favorable speech clarity in oral cavity reconstructions.

### 3.4. Mortality and Disease Progression

The overall mortality rate was 26.3% (*n* = 5) during the study period. Causes of death included systemic metastasis (*n* = 3), aspiration pneumonia (*n* = 1), and malignant pleural effusion (*n* = 1). Although three of the five deceased patients had experienced pharyngocutaneous fistula as a reconstruction-related complication, all deaths were primarily attributed to disease progression or systemic causes rather than direct reconstructive failure. These findings reflect the high-risk oncologic nature of the patient population.

* Representative Case 1: Maxillary Reconstruction with Chimeric TDAP Flap and Non-vascularized Bone Graft

**Figure 2:**
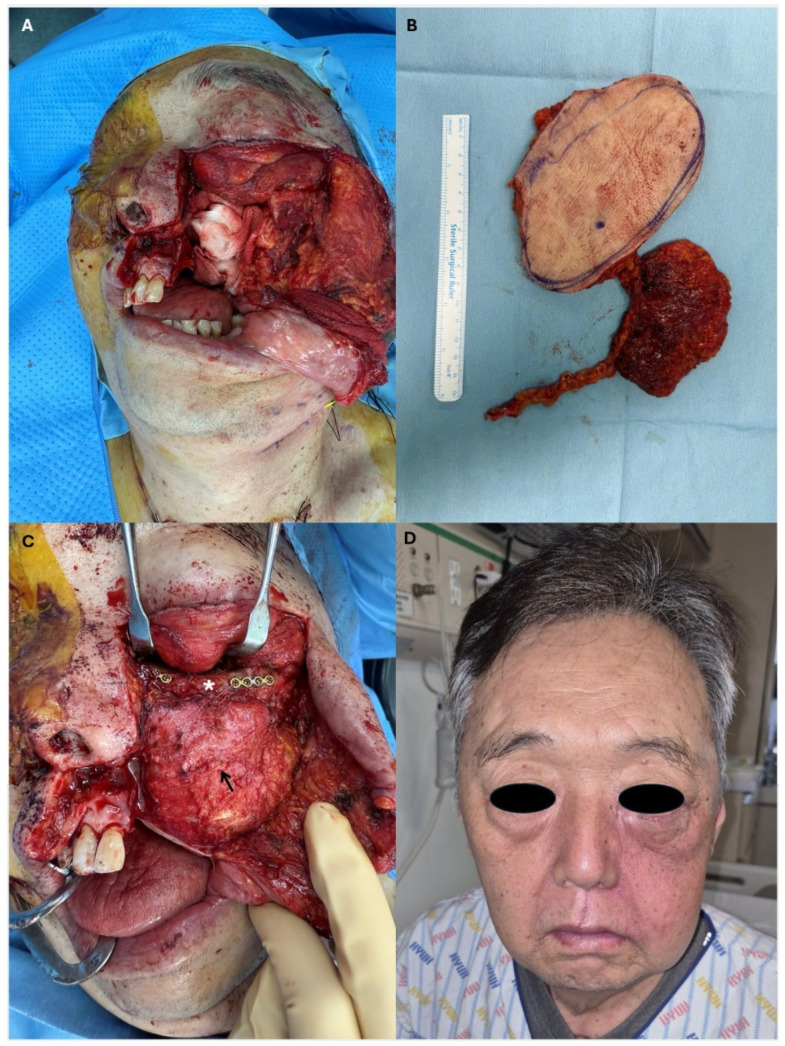
Chimeric TDAP flap for maxillary reconstruction. (**A**) Defect following total maxillectomy for high-grade mucoepidermoid carcinoma of the left maxillary sinus. (**B**) Harvested chimeric flap consisting of a TDAP skin paddle and partial latissimus dorsi muscle segment. (**C**) Flap inset. White asterisk indicates the iliac bone graft for orbital floor reconstruction; black arrow indicates the de-epithelialized skin paddle for nasal lining. (**D**) Postoperative appearance at 6 months after adjuvant chemoradiotherapy, showing satisfactory midface symmetry with mild lower eyelid ectropion.

A 65-year-old male presented with a high-grade mucoepidermoid carcinoma of the left maxillary sinus (pT3N0). The patient underwent total maxillectomy with selective neck dissection (levels II–III), resulting in a complex three-dimensional defect involving the orbital floor, maxillary sinus cavity, hard palate, and upper alveolus (Figure 2A).

The reconstructive goals were threefold: (1) restoration of orbital support to prevent enophthalmos, (2) obliteration of the maxillary sinus dead space, and (3) reconstruction of the oral lining to separate the oral and sinonasal cavities.

A chimeric flap based on the thoracodorsal system was designed. The flap incorporated a TDAP skin paddle (12 × 8 cm) and a partial latissimus dorsi muscle segment (8 × 6 cm), both perfused by a single thoracodorsal vascular pedicle (Figure 2B). For orbital floor reconstruction, a non-vascularized iliac bone graft was harvested and secured to the residual malar bone using titanium miniplates.

The flap was harvested in the supine position with a bolster placed under the right flank, enabling a simultaneous two-team approach. Microvascular anastomosis was performed to the facial artery and facial vein in an end-to-end fashion.

During flap inset, the LD muscle component was used to wrap the iliac bone graft, providing a vascularized biological envelope to promote bone graft incorporation. The muscle also served to obliterate the maxillary sinus dead space. A portion of the TDAP skin paddle was de-epithelialized and inset for nasal lining reconstruction (Figure 2C).

The patient achieved complete flap survival without fistula formation. Postoperative examination at 6 months, following adjuvant concurrent chemoradiotherapy, demonstrated satisfactory midface symmetry and stable orbital position. Mild left lower eyelid ectropion was noted, which was managed conservatively (Figure 2D).

* Representative Case 2: Pharyngolaryngeal Reconstruction with Three-Paddle Chimeric Flap

**Figure 3:**
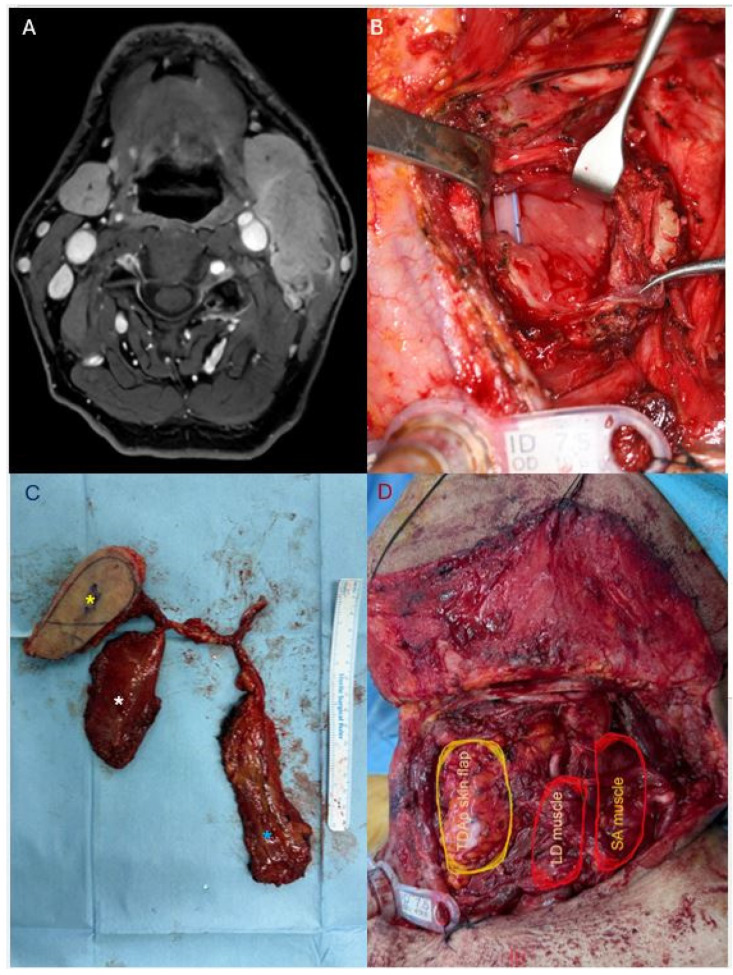
Three-paddle chimeric TDAP flap for pharyngolaryngeal reconstruction. (**A**) Preoperative MRI demonstrating left pyriform sinus tumor with metastatic lymphadenopathy showing extranodal extension, carotid artery abutment, and SCM muscle invasion. (**B**) Defect following partial laryngopharyngectomy and radical neck dissection, with exposed carotid artery and partial SCM resection. (**C**) Harvested three-paddle chimeric flap. Yellow asterisk: TDAP skin paddle; white asterisk: latissimus dorsi muscle; blue asterisk: serratus anterior muscle. (**D**) Flap inset. Yellow circle: TDAP skin paddle for pharyngeal mucosal reconstruction; red circles: LD muscle providing carotid artery coverage and SA muscle replacing the resected SCM.

A 69-year-old male presented with squamous cell carcinoma of the left pyriform sinus (pT1N3b). Preoperative MRI demonstrated a 2-cm enhancing lesion at the left pyriform sinus with suspected thyroid cartilage invasion. Metastatic lymphadenopathy at left level II–III showed extranodal extension with abutment to the carotid artery and invasion of the sternocleidomastoid (SCM) muscle (Figure 3A).

The patient underwent partial laryngopharyngectomy via transthyroid lateral pharyngotomy, radical neck dissection, partial parotidectomy, and submandibular gland excision. Intraoperatively, the tumor was found to invade the pyriform sinus with metastatic lymph nodes encasing the carotid artery and infiltrating the SCM muscle, necessitating partial SCM resection and exposure of the carotid artery (Figure 3B).

The reconstructive objectives were threefold: (1) restoration of pharyngeal mucosal continuity, (2) coverage of the exposed carotid artery to prevent blowout, and (3) reconstruction of the resected SCM muscle.

A three-paddle chimeric flap was designed based on the thoracodorsal system, incorporating a TDAP skin paddle (8 × 5 cm), a latissimus dorsi muscle segment (7 × 6 cm), and a serratus anterior muscle segment (11 × 5 cm) (Figure 3C). The flap was harvested in the supine position with a bolster under the right flank. Microvascular anastomosis was performed to the superior thyroid artery and external jugular vein in an end-to-end fashion.

The TDAP skin paddle was inset for pharyngeal mucosal reconstruction. The LD muscle was positioned to provide robust coverage over the exposed carotid artery. The SA muscle was used to reconstruct the partially resected SCM and obliterate the lateral neck dead space (Figure 3D).

The patient achieved complete flap survival without complications.

## 4. Discussion

This study suggests that the thoracodorsal artery-based chimeric flap system is a potentially useful option for complex head and neck reconstruction, achieving 100% flap survival in 19 consecutive cases. The following discussion addresses the rationale for this approach, practical surgical considerations, clinical versatility, and outcomes with limitations.

### 4.1. Rationale for the Thoracodorsal Chimeric System

Complex head and neck defects following oncologic ablation frequently demand simultaneous reconstruction of multiple tissue types—thin pliable lining, muscle bulk, external skin coverage, and occasionally bony framework—arranged in a three-dimensional spatial configuration [16]. While the double free flap approach can address these requirements, it substantially increases operative time, consumes additional recipient vessels, and imposes a significant physiological burden on a patient population that is often elderly and medically compromised [17,18].

The thoracodorsal chimeric flap offers an alternative by enabling multi-component reconstruction through a single donor site and a single set of microvascular anastomoses. A fasciocutaneous skin paddle, a latissimus dorsi muscle segment, and a serratus anterior muscle segment can each be independently designed and positioned, yet all share a common vascular supply. This eliminates the need for sequential flap procedures or prolonged operative times while preserving additional recipient vessels for potential future use.

### 4.2. Technical Considerations

#### 4.2.1. The Supine Approach and Surgical Efficiency

A practical but often underappreciated advantage of this technique is the feasibility of harvesting the thoracodorsal chimeric flap entirely in the supine position. By placing a small bolster beneath the ipsilateral flank, adequate exposure of the lateral chest and axilla can be achieved without formal lateral decubitus positioning. This enables a concurrent two-team approach, with the ablative and reconstructive teams working simultaneously. The resulting reduction in operative time is particularly valuable in elderly or medically compromised patients, who are overrepresented in head and neck oncology. The supine approach also simplifies anesthetic management by eliminating the hemodynamic fluctuations and airway concerns associated with intraoperative repositioning.

#### 4.2.2. Practical Considerations for Chimeric Flap Harvest

A legitimate concern for any reconstructive surgeon considering a chimeric flap is the additional technical demand of harvesting multiple tissue components. When a single free flap already requires considerable operative effort, the prospect of further dissection to incorporate a second or third component may appear disproportionate to the anticipated benefit.

In the authors’ experience, however, the thoracodorsal chimeric flap is technically more straightforward than one might anticipate. The thoracodorsal artery demonstrates highly consistent branching anatomy, with predictable bifurcation into descending and transverse branches and reliable angular branches to the serratus anterior muscle [13,19]. This anatomical predictability contrasts sharply with the ALT flap, where the number, caliber, and location of perforators are notoriously variable, rendering chimeric ALT configurations unpredictable and often unreliable [20]. With the thoracodorsal system, once the main pedicle is identified, the subsequent dissection to incorporate additional components follows a systematic and anatomically consistent course [12,19].

The advantage becomes more apparent when compared with alternative strategies for complex multi-component defects. Conjoint free flap configurations, which link two separate flaps through interposition vascular grafts, entail substantially greater technical complexity and operative risk [21]. Similarly, the intraoperative discovery of an inadequate pedicle length—necessitating vein grafting—introduces an additional anastomosis, prolongs operative time, and elevates the risk of thrombotic complications [22]. The thoracodorsal chimeric design avoids these scenarios entirely: all components share a common vascular origin, the mean pedicle length in this series was 7.5 cm, and no vein grafts were required.

In aggregate, the incremental effort required to harvest a chimeric configuration from the thoracodorsal system is modest relative to the reconstructive versatility gained and is substantially less than that demanded by the principal alternative strategies for multi-component defects.

#### 4.2.3. The Three-Paddle Configuration

The three-paddle chimeric configuration represents the most advanced application of this system. In one patient in this series requiring pharyngolaryngeal reconstruction, the TDAP skin paddle was used for pharyngeal mucosal restoration, the LD muscle for carotid artery coverage, and the SA muscle for sternocleidomastoid replacement—all through a single pedicle and single set of anastomoses. Achieving these three objectives with conventional approaches would have required either two separate free flaps or acceptance of compromised outcomes in one or more reconstructive domains. The independent mobility of each chimeric component permitted precise three-dimensional positioning without the geometric constraints inherent in single-paddle flap designs.

### 4.3. Clinical Versatility and Reconstructive Potential

#### 4.3.1. Multifunctional Role of Muscle Components

The muscle components of the thoracodorsal chimeric system serve multiple critical functions in head and neck reconstruction beyond simple soft tissue coverage. When positioned beneath the skin paddle, the muscle creates a two-layer closure that reinforces mucosal suture lines and provides a biological buttress against salivary leakage, thereby reducing the risk of pharyngocutaneous fistula [23]. The muscle bulk also obliterates dead space following radical ablation and neck dissection, mitigating the risk of seroma and hematoma formation [24]. In patients receiving adjuvant radiotherapy, the interposed muscle volume may buffer against radiation-induced contracture and volume loss [25]. Perhaps most critically, the muscle provides robust vascularized coverage of the carotid artery following radical neck dissection, creating a biological barrier against catastrophic blowout [26]. In cases where the sternocleidomastoid muscle is resected, the SA muscle, with its comparable bulk and shape, can be transposed into the defect to restore neck contour and provide a static soft tissue barrier over the great vessels. Although true functional reconstruction would require motor reinnervation, the volume replacement alone contributes to improved cosmetic outcome and structural protection.

It should be acknowledged that transplanted muscle undergoes progressive denervation atrophy over time, which may diminish its bulk and potentially compromise some of these functions in the long term [27]. However, the fibrotic scar tissue that replaces the atrophied muscle retains its role as a physical barrier—particularly for carotid artery protection and suture line reinforcement—and the initial volume provided during the critical early postoperative period, when the risks of fistula formation and carotid exposure are highest, remains a meaningful clinical contribution [28].

#### 4.3.2. Intraoperative Adaptability and Three-Dimensional Freedom

In the authors’ experience, one of the most clinically meaningful advantages of the thoracodorsal chimeric system is the degree of intraoperative freedom it affords the reconstructive surgeon. Head and neck defects are inherently unpredictable; the true extent and geometry of the defect are often not fully apparent until ablation is complete [29]. The chimeric configuration allows the surgeon to adjust the number, size, and spatial arrangement of flap components in real time, tailoring the reconstruction to the actual defect rather than committing to a preoperatively fixed design.

This adaptability has broader implications for reconstructive practice. Complex head and neck reconstruction has traditionally been constrained by the limited options available from a single flap—a constraint that often forces surgeons to choose between competing reconstructive objectives [17,30]. The thoracodorsal chimeric system, by providing multiple independently mobile tissue components through a single donor site, effectively expands the reconstructive repertoire available in a single operative setting. To provide a structured comparison of available reconstructive options for complex head and neck defects, the key characteristics of each approach are summarized in Table 3.

### 4.4. Outcomes, Complications, and Limitations

Functional recovery in this series was encouraging. All 14 surviving patients achieved tracheostomy decannulation and oral intake, with a mean time to oral feeding of 3.4 weeks. The thin, pliable nature of the TDAP skin paddle facilitated favorable mucosal adaptation in pharyngeal and oral cavity reconstructions.

Reconstruction-related complications occurred in 9 of 19 patients (47.4%), with pharyngocutaneous fistula or leakage being the most frequent (*n* = 6, 31.6%). This incidence is consistent with previously reported fistula rates of 15–40% in pharyngeal reconstruction, particularly in salvage settings [33,34]. When fistula occurred, conservative management was the initial approach, including wound care, nutritional optimization, and close observation. Surgical intervention was reserved for cases in which conservative measures failed to achieve spontaneous healing; in such instances, repair was performed using local flaps or rotation flaps. These complications were particularly prevalent in patients with prior radiation therapy, in whom tissue fibrosis and compromised vascularity increased the risk of fistula and delayed wound healing. In such cases, recipient vessels outside the irradiated field were selected (Figure 1), and a lower threshold for hospital admission and intravenous antibiotic therapy was maintained. Wound dehiscence occurred in two patients, both of whom had advanced-stage malignancy and poor general condition at the time of reconstruction, factors that may have adversely affected wound healing. The relatively high complication rate should therefore be interpreted in the context of advanced disease severity, as most patients presented with advanced head and neck malignancies accompanied by cachexia and nutritional compromise. Importantly, all complications were managed successfully without flap loss, supporting the reliability of the thoracodorsal vascular system in this high-risk population. The overall mortality rate of 26.3% reflected the substantial oncologic burden of this cohort. Although three of the five deceased patients had experienced pharyngocutaneous fistula, mortality was attributable to disease progression rather than reconstructive failure.

This reconstructive approach has inherent limitations. Donor-site morbidity includes measurable shoulder weakness and a visible scar. Flap bulk may be excessive when only minimal soft-tissue replacement is required. Prior axillary surgery may preclude flap harvest. Furthermore, the thoracodorsal chimeric system does not provide vascularized bone. In three cases in this series, non-vascularized bone grafts were used as adjuncts; however, when structural osseous reconstruction is the primary objective, a vascularized bone flap such as the fibula free flap remains the standard of care.

This study also has methodological limitations. Its retrospective design, extended study period (2009–2026), and limited follow-up in some patients introduce potential bias. All reconstructions were performed by a single senior surgeon across two institutions. While this ensured technical consistency and minimized inter-surgeon variability—an advantage in a technique-focused analysis—it may limit the external generalizability of the findings. The learning curve associated with chimeric flap harvest and multi-component inset may pose challenges for less experienced surgeons, and outcomes achieved in this series may not be readily reproducible in lower-volume settings without dedicated microsurgical training. Direct comparison with alternative reconstructive strategies was not undertaken, and objective functional assessments (e.g., VFSS, FEES) as well as patient-reported outcome measures were not systematically collected. Specifically, functional recovery was assessed by clinical milestones—tracheostomy decannulation and return to oral intake—rather than by standardized instruments such as the Functional Oral Intake Scale, validated speech intelligibility assessments, or patient-reported quality-of-life measures. Prospective comparative studies incorporating standardized functional and quality-of-life assessments are warranted to provide a more comprehensive evaluation of outcomes and reproducibility across different surgical settings.

## 5. Conclusions

The thoracodorsal artery-based chimeric flap system may represent a useful option for complex three-dimensional head and neck reconstruction, with 100% flap survival observed in this bi-institutional series of 19 patients. Its consistent vascular anatomy facilitates chimeric harvest, and the feasibility of supine positioning enable a simultaneous two-team approach. The capacity to independently orient multiple tissue components on a single pedicle provides intraoperative flexibility in addressing the heterogeneous geometry of advanced head and neck defects. Within appropriately selected cases requiring multi-component soft tissue reconstruction, the thoracodorsal chimeric flap may serve as an effective primary reconstructive strategy. However, the complication rate observed in this series underscores the complexity of reconstruction in this patient population, and further prospective studies are needed to validate these findings.

## Figures and Tables

**Table 1:** Patient Demographics and Clinical Characteristics (*n* = 19).

Variable	Value
Age (years), mean ± SD	63.2 ± 11.2
Sex (Male:Female)	14:5
Primary site	
Hypopharynx	8 (42.1%)
Oral cavity	7 (36.8%)
Others (larynx, maxilla, parotid)	4 (21.1%)
Flap configuration	
TDAP + LDm	11 (57.9%)
LDms + SAm	4 (21.1%)
TDAP + SAm	2 (10.5%)
LDmc + SAm	1 (5.3%)
TDAP + LDm + SAm	1 (5.3%)
Additional procedures	
Non-vascularized bone graft	3 (15.8%)
Supercharging	1 (5.3%)
Mean follow-up (months)	9.8 ± 8.3
Overall flap survival	19/19 (100%)
Overall mortality	5 (26.3%)
Reconstruction-related complications ^†^	9 (47.4%)
Pharyngocutaneous fistula/Leakage	6
Wound dehiscence	2
Seroma/Hematoma	2
Wound infection	1

^†^ Two patients experienced multiple complications; total complication events = 11. Abbreviations: TDAP, thoracodorsal artery perforator skin paddle; LDm, latissimus dorsi muscle; LDms, latissimus dorsi muscle-sparing skin paddle; LDmc, latissimus dorsi musculocutaneous flap; SAm, serratus anterior muscle.

**Table 2:** Summary of 19 Cases Using Thoracodorsal Artery-Based Chimeric Flaps.

Case	Age/Sex	Diagnosis	Flap Configuration	Bone Graft	Complication	Outcome
1	60/M	Recurrent glottic SCC	TDAP + LDm	—	Fistula	Expired
2	54/M	Hypopharyngeal SCC	TDAP + LDm	—	—	Survived
3	60/M	Hypopharyngeal SCC	TDAP + LDm	—	Infection	Survived
4	57/M	Hypopharyngeal SCC	TDAP + LDm	—	—	Survived
5	58/M	Hypopharyngeal SCC	TDAP + LDm	—	Fistula	Expired
6	55/M	Hypopharyngeal SCC	TDAP + LDm	—	Fistula	Expired
7	78/M	Tongue base SCC	TDAP + LDm	—	—	Survived
8	42/M	Parotid carcinoma	TDAP + SAm	—	—	Expired
9	54/F	Buccal SCC	LDms + SAm	—	Fistula	Survived
10	50/M	Tongue base SCC	TDAP + LDm	—	Hematoma	Survived
11	75/F	Oral cavity SCC	LDms + SAm	—	Dehiscence, Fistula	Survived
12	72/F	Gingivobuccal SCC	LDmc + SAm	NV fibula	—	Expired
13	69/M	Hypopharyngeal SCC	LDms + SAm	—	—	Survived
14	65/M	Maxillary sinus MEC	TDAP + LDm	NV iliac bone	—	Survived
15	80/F	Tongue base SCC	TDAP + LDm	—	Seroma	Survived
16	82/F	Alveolar SCC	LDms + SAm	NV fibula	—	Survived
17	69/M	Glottic SCC	TDAP + LDm	—	—	Survived
18	51/M	Hypopharyngeal SCC	TDAP + SAm	—	Dehiscence, Leakage	Survived
19	69/M	Hypopharyngeal SCC	TDAP + LDm + SAm	—	—	Survived

Abbreviations: TDAP, thoracodorsal artery perforator skin paddle; LDm, latissimus dorsi muscle; LDms, latissimus dorsi muscle-sparing flap; LDmc, latissimus dorsi musculocutaneous flap; SAm, serratus anterior muscle; SCC, squamous cell carcinoma; MEC, mucoepidermoid carcinoma; NV, non-vascularized.

**Table 3:** Comparison of reconstructive options for complex head and neck defects.

Flap Type	Tissue Components	Pedicle Length	3D Versatility	Key Advantages	Key Limitations
ALT [8]	Skin, fascia, (±vastus lateralis muscle)	8–12 cm	Moderate	Long pedicle; low donor morbidity; large skin paddle	Highly variable perforator anatomy; limited to single-layer reconstruction without additional flap
Radial forearm [31]	Skin, fascia	Up to 20 cm	Low	Thin, pliable tissue; long pedicle; reliable anatomy	Single tissue type; sacrifices major forearm artery; limited bulk
Fibula [32]	Bone, skin paddle	4–6 cm	Moderate	Vascularized bone for mandibular reconstruction; allows dental rehabilitation	Soft tissue component often insufficient for mucosal and skin coverage; short pedicle
Pectoralis major (pedicled) [26]	Skin, muscle	N/A	Low	No microsurgery required; reliable blood supply; rapid harvest	Bulky; limited reach; poor tissue match for mucosal lining; aesthetic donor-site deformity
Double free flap [6,17,18]	Variable	Variable	High	Optimal flap selection for each subunit; flexible positioning; independent vascular supply	Prolonged operative time; two sets of anastomoses; consumes multiple recipient vessels; increased physiological burden
TDA chimeric (present study)	TDAP skin paddle, LD muscle, SA muscle, (±non-vascularized bone)	7–8 cm	High	Multiple independently positionable components; consistent vascular anatomy; single pedicle; true 3D reconstruction	Donor-site morbidity (shoulder weakness, scar); potentially excessive bulk; prior axillary surgery may preclude harvest

ALT = anterolateral thigh; TDA = thoracodorsal artery; TDAP = thoracodorsal artery perforator; LD = latissimus dorsi; SA = serratus anterior; 3D = three-dimensional.

## Data Availability

The data presented in this study are not publicly available due to patient privacy restrictions under institutional regulations and Korean law. Anonymized summary data are presented within the article.

## Abbreviations

The following abbreviations are used in this manuscript:

TDAP	Thoracodorsal artery perforator
LD	Latissimus dorsi
SA	Serratus anterior
ALT	Anterolateral thigh
SCM	Sternocleidomastoid
